# Surveillance of respiratory viruses by aerosol screening in indoor air as an early warning system for epidemics

**DOI:** 10.1111/1758-2229.13303

**Published:** 2024-07-09

**Authors:** Zeynep Bengi Eren, Cansel Vatansever, Berk Kabadayı, Bedirhan Haykar, Zeynep Ece Kuloğlu, Sedat Ay, Kamila Nurlybayeva, Gül Eyikudamacı, Tayfun Barlas, Erhan Palaoğlu, Yeşim Beşli, Mert Ahmet Kuşkucu, Önder Ergönül, Fusun Can

**Affiliations:** ^1^ Koç University School of Medicine Istanbul Turkey; ^2^ Koç University İşBank Center for Infectious Diseases (KUISCID) Istanbul Turkey; ^3^ Koç University Graduate School of Health Sciences Istanbul Turkey; ^4^ Department of Clinical Laboratory American Hospital Istanbul Turkey; ^5^ Department of Medical Microbiology Koç University School of Medicine Istanbul Turkey; ^6^ Department of Infectious Disease and Clinical Microbiology Koç University School of Medicine Istanbul Turkey

## Abstract

The development of effective methods for the surveillance of seasonal respiratory viruses is required for the timely management of outbreaks. We aimed to survey Influenza‐A, Influenza‐B, RSV‐A, Rhinovirus and SARS‐CoV‐2 surveillance in a tertiary hospital and a campus over 5 months. The effectiveness of air screening as an early warning system for respiratory viruses was evaluated in correlation with respiratory tract panel test results. The overall viral positivity was higher on the campus than in the hospital (55.0% vs. 38.0%). Influenza A was the most prevalent pathogen in both locations. There were two influenza peaks (42nd and 49th weeks) in the hospital air, and a delayed peak was detected on campus in the 1st‐week of January. Panel tests indicated a high rate of Influenza A in late December. RSV‐A‐positivity was higher on the campus than the hospital (21.6% vs. 7.4%). Moreover, we detected two RSV‐A peaks in the campus air (48th and 51st weeks) but only one peak in the hospital and panel tests (week 49). Although rhinovirus was the most common pathogen in panel tests, rhinovirus positivity was low in air samples. The air screening for Influenza‐B and SARS‐Cov‐2 revealed comparable positivity rates with panel tests. Air screening can be integrated into surveillance programs to support infection control programs for potential epidemics of respiratory virus infections except for rhinoviruses.

## Graphical abstract

Development of effective methods for surveillance of seasonal respiratory viruses is required for timely management of outbreaks. The aim of the study was to survey respiratory viruses' surveillance in a tertiary hospital and a campus over a five‐month period. We concluded that air screening can be integrated into surveillance programs to identify and support infection control programs for potential epidemics of respiratory virus infections except rhinoviruses.

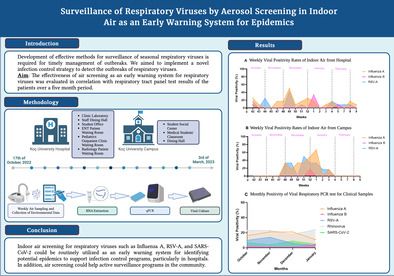

## INTRODUCTION

Viral respiratory infections are a global public health problem that causes significant illness and death, especially among vulnerable populations such as young children, the elderly, those with chronic diseases, and immunocompromised patients (Cilloniz et al., 2022; Greenberg, 2002). Respiratory viruses typically spread through the air, mainly through droplets or aerosols (Hodinka, 2016). In 2021, the World Health Organization (WHO) and the US Centers for Disease Control and Prevention (CDC) officially recognized the inhalation of virus‐laden aerosols as the primary mode of SARS‐CoV‐2 transmission, both at short and long distances (CDC, 2023a; WHO, 2023). It has also been confirmed that influenza virus, respiratory syncytial virus (RSV), human rhinovirus (HRV), and severe acute respiratory syndrome coronavirus (SARS‐CoV) can be transmitted through aerosols (Raymenants et al., 2023; Wang et al., 2021).

The transmission of aerosols usually occurs indoors. During the COVID‐19 pandemic, the monitoring of aerosols in the indoor air has become increasingly crucial, particularly within healthcare institutions like hospitals. Recent studies showed that SARS‐CoV‐2 bioaerosol load in the indoor air of the hospital was higher compared to outdoor air in the hospital and indoor air in community settings (Alsved et al., 2023; Dinoi et al., 2022). Aerosol screening in the community setting also drew attention in the same period. Raymenantst et al. reported 85.3% positivity for at least one of the 29 respiratory pathogens in indoor air samples from 21 community settings (Raymenants et al., 2023).

The risk of transmission through aerosols depends on various factors related to the building, the type of pathogens circulating, the number of people present, and their behaviours such as wearing masks, laughing, speaking loudly, sneezing, or coughing (Raymenants et al., 2023). Besides, social distancing measures have been identified as critical factors in mitigating the spread of the virus during the COVID‐19 pandemic period (Kim et al., 2020). Furthermore, it was reported that viral spread was strongly influenced by the age of people present in the location due to different social behaviours between age groups (Kucharski et al., 2014).

Recent studies have also provided evidence linking aerosols to outbreaks in small and crowded environments such as classrooms, churches, and restaurants (Buonanno et al., 2022; Miller et al., 2021). High concentrations of aerosols containing the influenza‐A virus were detected at various locations on a university campus during an influenza‐A outbreak (Ramuta et al., 2022). The surveillance data revealed a difference in the timing and location of SARS‐CoV‐2 and influenza A virus detection. Specifically, while Influenza A virus detection correlated with a localized outbreak on the campus, SARS‐CoV‐2 transmission exhibited a broader spread, evident across various testing sites within the community (Ramuta et al., 2022).

In addition to quantifying pathogens and assessing the risk of transmission in indoor environments, the implementation of active surveillance programs is crucial for outbreak prevention. Ambient air monitoring was used during the COVID‐19 pandemic (Kayalar et al., 2021). However, the effectiveness of air monitoring for respiratory virus surveillance has not yet been validated. Currently, various air collection systems are employed across diverse settings, each with its characteristics in timing and airflow rate for virus detection (Lin et al., 2018; Pan et al., 2019). Recent studies emphasize the efficiency of the systems with high‐speed air collection into rotating fluid tubes (Kuloğlu et al., 2023).

Changes in the epidemiology of RSV‐A and Influenza‐A viruses after the COVID‐19 pandemic have increased the importance of active surveillance for respiratory viruses (McNab et al., 2021; NREVSS, 2023). Thus, the implementation of proactive surveillance strategies is essential for mitigating future outbreaks and safeguarding public health.

We aimed to detect Influenza A, Influenza B, RSV‐A, Rhinovirus, and SARS‐CoV‐2 viruses in aerosols in indoor air during the peak season and assess the usefulness of aerosol screening for epidemiological surveillance of respiratory pathogens in the community and hospital.

## EXPERIMENTAL PROCEDURES

### Study design and air sampling

This study was conducted in a tertiary university hospital and main university campus in Istanbul, Turkey. The indoor aerosol samples were collected during the peak season of respiratory infections between the 17 October 2022, and the 3 March 2023. The air samplings were done using the Coriolis μ‐biological air sampler (Bertin Instruments, France) with a flow rate of 200 L/min for 30 min according to manufacturer instructions and previous studies (Papin et al., 2005; Kapmaz et al., 2023; Kuloğlu et al., 2023). Briefly, more than 30 min of continuous sampling with Coriolis μ Air lead to inconsistent sample recovery from the collector cone (Rufino de Sousa et al., 2020). Before starting sample collection, we did pilot studies to set the optimum time and airflow rate (Kuloğlu et al., 2023). We placed the device at the same point in each sample collection process. Aerosol samples were collected in 10 mL of DMEM High‐Glucose (Sigma‐Aldrich®, cat. no: D6429) supplemented with 1% Penicillin–Streptomycin (HyClone™, cat. no: SV30010) and Amphotericin B (HyClone™, cat. no: SV30078.01). During the sampling, the number of people present at the time of collection and the dimensions of the sampling area were recorded. The crowding index was calculated by the average number of people divided by the volume (m^3^). In the same period, the results of the respiratory virus panel test for Influenza A, Influenza B, Sars‐CoV‐2, and RSV‐A and Rhinovirus detection (Roche, Basel, Switzerland) and the card tests for Influenza A, Influenza B, and RSV‐A diagnosis which were performed on patients admitted to the clinical microbiology laboratory in the hospital were also recorded.

### Viral studies

Vero E6 Cells (ATCC, Manassas, VA, USA; No. CRL‐1586), MDCK‐1 (ATCC, Manassas, VA, USA; No. NR‐2628), and A549 cell lines (ATCC, Manassas, VA, USA; No: CCL‐185) were cultured in flasks with DMEM high glucose (Sigma, St. Louis, MO, USA) supplemented with 10% foetal bovine serum (Gibco; ThermoFisher Scientific, Waltham, MA, USA), 1% penicillin–streptomycin (Hyclone; Cytiva Life Sciences, Marlborough, MA, USA), and amphotericin B (Hyclone). Upon reaching confluency, MDCK‐1 cells were infected with Influenza A and Influenza B viruses, Vero E6 cells were infected with SARS‐CoV‐2 and Rhinovirus, and A549 cells were infected with RSV (Lugovtsev et al., 2013; Ogando et al., 2020; Saleh et al., 2020; van den Braak et al., 2022). The supernatant was collected upon visible cytopathic effect (CPE). Viral RNA was extracted using the EZNA Viral RNA Kit (cat no: R6874, Omega, Norcross, Georgia, US) following the manufacturer's instructions.

The RNA stock of Influenza A, Influenza B, SARS‐CoV‐2, RSV, and Rhinovirus served as positive controls for Quantitative Reverse Transcriptase PCR (qRT‐PCR) after confirmation of positivity with specific primers. Briefly, cDNA synthesis was performed using the iScript Biorad cDNA kit, followed by qRT‐PCR with primers using the QuantStudio 7 Flex Real‐Time PCR System (Applied Biosystems, ThermoFisher Scientific). All experiments were performed in duplicates with three biological replicates.

For air samples, viral RNA was extracted from collected samples in DMEM using the E.Z.N.A. Viral RNA Kit. Subsequently, cDNA synthesis was conducted using the iScript Biorad cDNA kit, followed by qRT‐PCR with primers using the QuantStudio 7 Flex Real‐Time PCR System (CDC, 2021; Kuloğlu et al., 2023; Liu et al., 2020; Lopez et al., 2021). Positive controls were included using RNA stocks of Influenza A, Influenza B, SARS‐CoV‐2, RSV, and Rhinovirus. Also, nuclease‐free water was used as a negative control in qRT‐PCR. Threshold cycle values (Ct) were analysed. qPCR‐positive air samples were inoculated in triplicates on appropriate cells for each virus in 96‐well plates with 90%–100% confluency. Cells were also infected with PBS to be used as a negative control. Cytopathic effects were monitored on day 6, and culture supernatants were collected on day 7. Viral growth was confirmed with qRT‐PCR from culture supernatants showing visible cytopathic effects under the inverted microscope. The threshold Ct for positivity was 36. All experiments were performed in duplicates with three biological replicates.

## RESULTS

A total of 276 samples were collected in the hospital (*n* = 216) and the campus (*n* = 60). In the campus, 55.0% of the indoor air samples were positive for at least one respiratory virus RNA. The highest rate of positivity, 6.5%, was found in the University Student Social Center, followed by the University Dining Hall at 5.5%. In the hospital, the overall viral RNA positivity rate was 38.0%. The Radiology Patient Waiting Room had the highest positivity rate at 8.3%, and the second highest viral RNA positivity rate, 6.6%, was detected in the Staff Dining Hall (Table 1). The crowding index was higher in all areas of the hospital compared to the campus areas. A weak correlation (*R* = 0.39) was found between the average population in the sampling areas and the total viral positivity.

**TABLE 1:** The positivity of respiratory viruses in indoor air of different areas in campus and hospital.

Main area	Locations	Total positivity (%)	Influenza A positivity (%)	RSV‐A positivity (%)	SARS‐CoV‐2 positivity (%)	Influenza B positivity (%)	Human rhinovirus RNA positivity (%)	Mean, *C*_t_	Room temp.	Mask use^a^	Crowding index^b^
Campus	University student social centre	13.00% (13/100)	30.00% (6/20)	30.00% (6/20)	0.00%	5.00% (1/20)	0.00%	32,835	23.5	No	0.02387775
	University dining hall	11.00% (11/100)	25.00% (5/20)	30.00% (6/20)	0.00%	0.00%	0.00%	32,28	23.5	No	0.02970297
	Medical student classroom	5.00% (5/100)	20.00% (4/20)	5.00% (1/20)	0.00%	0.00%	0.00%	34,402	22.5	No	0.0494382
Hospital	Radiology patient waiting room	8.30% (15/180)	22.20% (8/36)	8.30% (3/36)	2.80% (1/36)	5.60% (2/36)	2.80% (1/36)	34,67	22.1	Yes	0.22962963
	Staff dining hall	6.60% (12/180)	11.10% (4/36)	13.90% (5/36)	8.30% (3/36)	0.00%	0.00%	31,426	22.1	No	0.25806452
	Clinical laboratory patient waiting room	6.10% (11/180)	13.90% (5/36)	8.30% (3/36)	2.80% (1/36)	2.80% (1/36)	2.80% (1/36)	31,382	22.1	Yes	0.18253968
	Student office	4.40% (8/180)	16.70% (6/36)	2.80% (1/36)	0.00%	2.80% (1/36)	0.00%	34,562	22.1	No	0.03162055
	Paediatrics outpatient clinic waiting room	3.30% (6/180)	8.30% (3/36)	5.60% (2/36)	0.00%	2.80% (1/36)	0.00%	32,907	22.1	Yes	0.09545455
	Ear nose throat patient waiting room	1.70% (3/180)	2.80% (1/36)	5.60% (2/36)	0.00%	0.00%	0.00%	33,582	22.1	Yes	0.1080402

*Note*: Colour shade represents the positivity ratio rates. Hence, darker regions show a higher positivity compared to lighter shaded regions.

^a^ The Turkish Ministry of Health has announced the end of mask requirements in all closed areas, except public transportation and healthcare institutions starting 27 April 2022.

^b^ The crowding index was calculated by the average number of people divided by the volume (m^3^).

Influenza A was the most common pathogen on the campus (25%) and in the hospital indoor air (12.5%). The positivity rate for RSV‐A was also higher on the campus (21.6%) compared to the hospital (7.4%). In addition, the positivity rate of RSV‐A increased to 30% in the Student Social Center and Student Dining Hall on campus. In the hospital. RSV‐A positivity was found to be very low (5.6%) in the Paediatrics Outpatient Clinic Waiting Room while the highest rate was measured in the Staff Dining Hall (13.9%). Rhinovirus and SARS‐CoV‐2 positivity rates were low in the hospital, and these viruses were not detected on the campus (Table 1).

**FIGURE 1:**
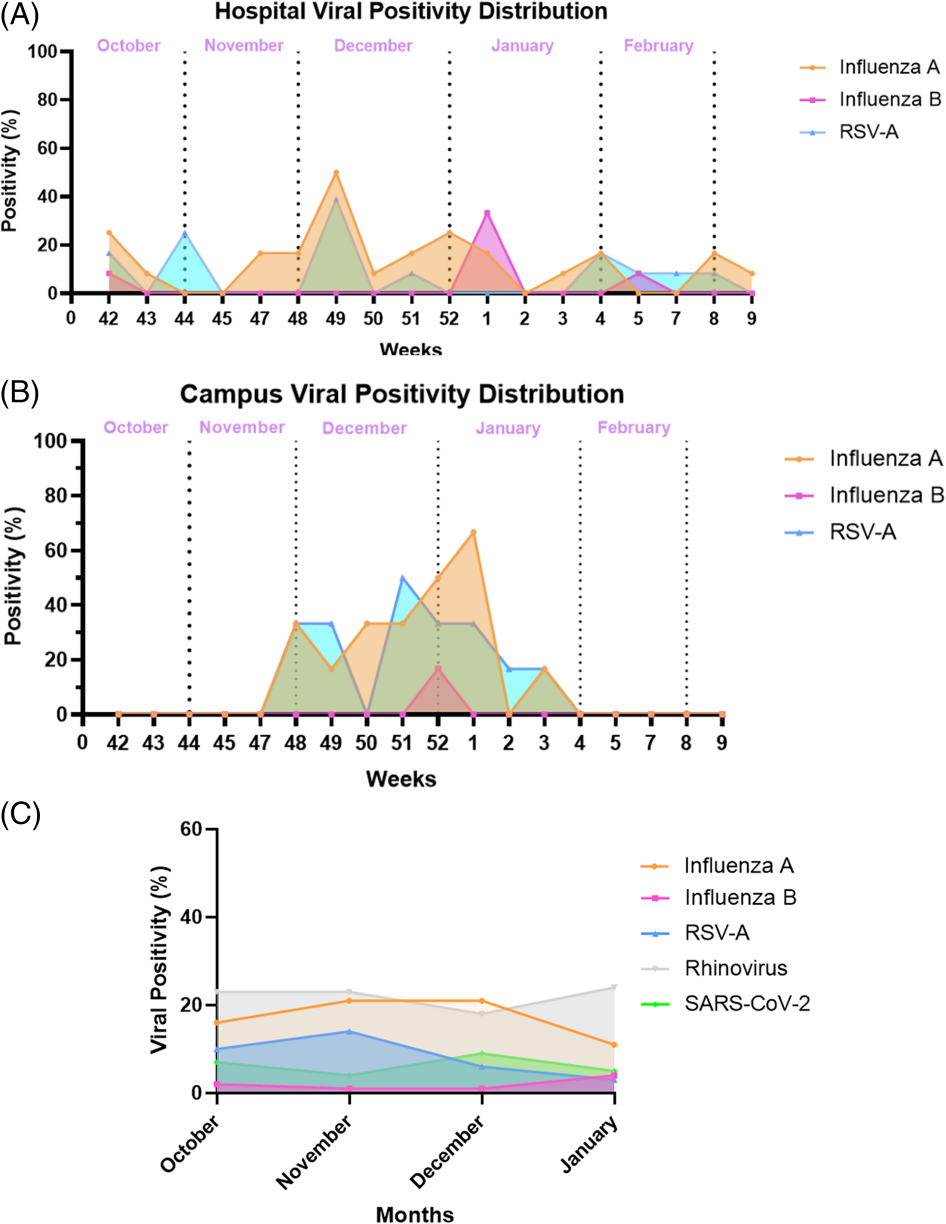
The Viral positivity distribution in indoor environment in campus and hospital across time. (A) Weekly viral positivity rates of indoor air from the Hospital. Each week's number is attributed with respect to the corresponding week in the calendar. (B) Weekly viral positivity rates of indoor air from Campus. Each week's number is attributed with respect to the corresponding week in the calendar. (C) Monthly positivity of viral respiratory PCR test for clinical samples.

The temporal variations of all pathogens in indoor air were similar in the hospital and campus, exhibiting two peaks. In the campus, the first peak was observed during weeks 48–49, followed by a second peak during weeks 52–1, with positivity rates of 83.3% and 100% respectively. In the same period, the hospital had positivity rates of 75% and 50% (Figure 1A, B).

In the hospital, the rate of RSV‐A positivity increased to a peak level of 41.7% in week 49 and remained below 25% during the study period (Figure 1A). The results of the RSV‐A tests for clinical samples were consistent with the aerosol results, with the highest percentage being 15% in November (Figure 1C). In the Campus, RSV‐A had two peaks, the first was in week 48 with a positivity rate of 33.3% followed by 50% positivity in week 51 (Figure 1B).

Influenza A had a small peak (week 42; 25%) in the hospital air, and the highest peak was observed in early December (week 49; 50%). On campus, there was a four‐week delay for the peak of influenza A (66.7%), which was detected in the first week of January. Thereafter, positivity rates decreased below 20% at both locations. We did not start screening on Campus in October. Similar to the aerosol data from Campus, the results of clinical laboratory tests showed a high rate of Influenza A positivity in December (21%), with a decrease to 11% in January (Figure 1C). During the study period, influenza B in the air became positive in late December with rates of 16.7% and 33.3% in Campus and Hospital, respectively. Likewise, the results of clinical tests for Influenza B showed a similar trend to indoor air rates.

We detected SARS‐CoV‐2 in hospital indoor air at week 51 (16.6%). SARS‐CoV‐2 positivity rates of clinical samples were also high over the same period. None of the air samples were positive on Campus.

Although the Rhinovirus was the most common pathogen found in clinical samples, the PCR results from indoor air in the hospital and campus were not consistent with the clinical test results. We were able to detect Rhinovirus in the hospital air with a positivity rate of 8.3%, while the positivity rate in patient samples was high (between 18% and 50%). There was no Rhinovirus positivity in the campus air.

Among 90 PCR‐positive air samples, two samples (2.22%) were grown in the cell culture. Influenza B was grown in the hospital student office air (qPCR Ct 35.790) and another air sample was from the paediatric outpatient waiting room with RSV‐A culture positivity (qPCR Ct 36.009).

## DISCUSSION

The COVID‐19 pandemic has caused significant changes in the epidemiology of respiratory viruses, especially for RSV‐A and Influenza‐A viruses. Previous studies have shown that the detection of viral genome by qPCR on ambient air can be utilized as a tool for monitoring respiratory pathogens (Raymenants et al., 2023). However, the effectiveness of air screening depends on many factors such as the behaviour of the people in the environment, building‐related differences, and technical specifications (Ramuta et al., 2022). In this study, we monitored five respiratory viruses in indoor air over 5 months in a tertiary hospital and on a university campus. We found a higher positivity of respiratory pathogens in the air from the campus (55.0%) compared to the hospital (38.0%). Despite more crowded and possibly infected populations gathered in the hospital, the lower positivity rate of viral pathogens can be explained by the HEPA‐filtered air conditioning systems and mandatory use of masks in the hospital during the study period. Universal masking has proven to be an efficient and cost‐effective measure in preventing the spread of virus‐laden aerosols (Tanisali et al., 2021). The transmission of the virus in the air is also associated with attendee behaviours such as talking, laughing, and eating. The high viral positivity rates in the Student Social Center (6.5%) and the Student Dining Hall (5.5%) on campus support previous reports that highlight the increased risk of viral spread through the air with certain active behaviours like laughing, and speaking loudly. Similarly, the positivity of the Influenza A virus was found to be lower in classrooms (1900 gene copies m^3^ air)compared to corridors (19,000 gene copies m^3^ air) in an elementary school (Coleman, 2020). We did not evaluate factors related to building design.

Influenza A (25.0% campus and 12.5% hospital) and RSV‐A (21.7% campus and 7.4% hospital) were the most frequently identified in both locations. Respiratory viral infections season usually starts in October till March each year. According to the CDC report of Influenza Disease Burden in the season of 2021–2022, Influenza accounts for 9.4 million flu illnesses, 4.3 million flu‐related medical illnesses, and 100,000 flu‐related hospitalizations between November 2022 and January 2022 (CDC, 2023b). However, the epidemiology of respiratory viruses significantly changed after the COVID‐19 pandemic (Falsey et al., 2022; McNab et al., 2021). During the pandemic period, there was a significant reduction in RSV and Influenza cases (Falsey et al., 2022; Munkstrup et al., 2023). However, surveillance reports have pointed out that RSV and influenza cases increased in the post‐pandemic period (McNab et al., 2021). According to the Ministry of Health surveillance report in Turkey, RSV (5.6.%) and Influenza (30.1%) peaked in the 51st week of 2022 with values significantly higher than in previous years (CDC, 2022; CDC, 2023c). The peak levels of these viruses in the Campus air were in line with the Ministry of Health report, which were seen in the 51st and 1st weeks. However, in the hospital air, we detected the highest peak 4 weeks earlier (week 49) than the Campus. In addition, there was another influenza A peak which was seen in October. The rate of influenza infections among children was higher than among adults (Ruf & Knuf, 2014). In our hospital, we collected air from the paediatric outpatient clinic. The early peak in the hospital air could be due to the paediatric patient population in the hospital. The influenza A PCR positivity from nasopharyngeal specimens in our study was also parallel with the aerosol positivity in the hospital. Influenza A tests become positive in December. The sensitive detection of influenza A in air samples suggests that air screening can be adapted to hospital surveillance programs to determine the optimal timing for vaccination of healthcare workers as a part of infection control programs. The surveillance of influenza infections in hospitals is gaining more importance since the proportion of hospital‐acquired infections among influenza cases increased in the last decade with high geographic differences, from 0.3% in Italy to 35.53% in Germany (Amodio et al., 2014; Rossler et al., 2021). The high influenza vaccination coverage among healthcare workers significantly reduces the incidence rate of HIA among patients (Riphagen‐Dalhuisen et al., 2013). Moreover, the highest positivity of influenza A in the radiology unit (22.2%) is also an important finding due to the high diversity of the patient population in this department (immunocompromised, elderly, infected, etc.). Therefore, we suggest that air screening could be useful for identifying hospital departments requiring extra precautions.

RSV is commonly considered a childhood disease and causes outbreaks among children, particularly in the autumn‐winter season (Keske et al., 2022). However, we found that the positivity of RSV‐A in the air of the Paediatric outpatient clinic was low (5.6%), while it was 13.9% in the Staff Dining Hall, where only adults gathered. The detection of the first peak of RVS in both hospital air and clinical samples in week 44 suggested to us that air sampling could also be used for RSV monitorization. Furthermore, we observed a significant high positivity of RSV‐A on Campus (21.7%), especially towards the end of the year when students have gatherings to celebrate the new year. Various studies from different regions have reported increased cases of RSV‐A in the post‐pandemic period, highlighting a change in the epidemiology of RSV‐A infections with an increase in cases among adults (CDC, 2023a; Maglione et al., 2022; McNab et al., 2021). In 2021, the United States experienced a sudden and significant surge in RSV cases during the interseasonal period (Falsey et al., 2022). In autumn 2022, Denmark experienced an RSV epidemic, which was unusual for that time of year with the dominance of subtype B (Munkstrup et al., 2023). Considering all the reports, it is evident that active surveillance in the community is necessary to fully comprehend the extent and nature of these shifts in RSV‐A epidemiology. Our data suggest that screening for RSV‐A virus in the indoor air of public places, and large workspaces such as factories is particularly important for the prediction of possible outbreaks and to decide whether to implement preventive measures such as RSV‐A vaccine administration to students and employees.

Despite the high rhinovirus positivity observed in our clinical laboratory tests, the low proportion of rhinovirus in air samples was probably due to technical limitations. We utilized the Coriolis μ‐biological air sampler at a flow rate of 200 L/min. The sampler type, humidity flow rate, duration of sampling, and the volume of the area can all influence pathogen detection (Raymenants et al., 2023). One of the limitations of our study we did not record humidity during air sampling. In previous studies, the Coriolis sampler was used to detect SARS‐CoV‐2 in the air, but there was no report of rhinovirus (Gulraiz et al., 2014; Kuloğlu et al., 2023; Zhou et al., 2023). It is unclear why the air samples detected little rhinovirus, despite this virus being more commonly detected in clinical samples (Huang et al., 2021; Sullivan et al., 2020; Tang et al., 2021). Since we did not test another method for rhinovirus screening in the air, we could not suggest another method.

In this study, two indoor air samples yielded viral growth. This could be due to the low viral load in the air samples or damage that could have occurred to the viral particles during the air sampling. The low correlation between RNA levels and the cell culture growth rate of the Omicron variant was reported (Puhach et al., 2022).

In conclusion, indoor air screening for respiratory viruses such as Influenza A, RSV‐A, and SARS‐CoV‐2 could be routinely utilized as an early warning system for identifying potential epidemics to support infection control programs, particularly in hospitals. In addition, air screening could help active surveillance programs in the community since this approach is feasible, cost‐effective, and supports large‐scale screening of the populations. However, extra validation studies are necessary before the introduction of air screening systems in the routine screening protocols.

## AUTHOR CONTRIBUTIONS

**Zeynep Bengi Eren:** Conceptualization; writing – original draft; methodology; visualization; project administration; funding acquisition. **Cansel Vatansever:** Writing – original draft; methodology; conceptualization. **Berk Kabadayı:** Methodology; visualization; writing – original draft. **Bedirhan Haykar:** Methodology; visualization. **Zeynep Ece Kuloğlu:** Methodology; visualization. **Sedat Ay:** Methodology. **Kamila Nurlybayeva:** Methodology. **Gül Eyikudamacı:** Methodology. **Tayfun Barlas:** Methodology; resources. **Erhan Palaoğlu:** Methodology; validation. **Yeşim Beşli:** Methodology; validation. **Mert Ahmet Kuşkucu:** Methodology; conceptualization. **Önder Ergönül:** Conceptualization; validation; visualization; writing – original draft. **Fusun Can:** Conceptualization; funding acquisition; writing – original draft; methodology; validation; visualization.

## CONFLICT OF INTEREST STATEMENT

The authors declare no conflicts of interest.

## ETHICS STATEMENT

The study was approved by the Koç University Ethical Committee with decision number 2022.057.IRB.056 on 10 February 2022.

## Data Availability

The raw data that support the findings of this study are available on request from the corresponding author, F.C.
